# Thalamic Bursts and the Epic Pain Model

**DOI:** 10.3389/fncom.2016.00147

**Published:** 2017-01-12

**Authors:** Carl Y. Saab, Lisa Feldman Barrett

**Affiliations:** ^1^Department of Neurosurgery, Rhode Island HospitalProvidence, RI, USA; ^2^Department of Neuroscience, Brown UniversityProvidence, RI, USA; ^3^Department of Psychology, Northeastern UniversityBoston, MA, USA

**Keywords:** burst, thalamus, pain, cortex, predictive coding

## Introduction

Multiple nociceptive pathways in the nervous system have been identified based on structural connectivity studies (Willis, 1991, 2007). However, scientific understanding of the neural dynamics underlying traffic patterns along these highways (i.e., functional connectivity) remains incomplete. Extant techniques for sampling neural data at the micro-scale level using single-unit electrophysiology, and at the macro-scale level using whole brain fMRI, have left a wide knowledge gap at the level of mesoscale network dynamics mediating pain perception.

Approaches involving neural ensemble recordings combined with optogenetic interventions represent a valid strategy for closing this knowledge gap. However, significant research efforts to simply amass big data without a valid conceptual framework risk being misguided; data without theory are just numbers. We propose a unifying framework for the interpretation of neural data at the cellular and network levels, generating testable hypotheses, and leading to a more comprehensive understanding of pain in the context of network dynamics.

## Pain: a national health crisis

Healthcare providers and several branches of government are promoting research initiatives that improve our understanding of the cellular mechanisms of pain. Pain affects a third of the U.S. population with healthcare costs exceeding $600 billions per year (Academies, 2011). On average, it takes a patient with chronic pain 12 years and more than five referrals to be admitted to a specialized pain center (Schulte et al., 2010). Pharmacotherapy remains suboptimal, especially in the face of high placebo effects, while most prescription painkillers cause significant side effects such as addiction and lethal overdose.

## An EPIC pain framework

Extensive studies based on the structural connectivity of nociceptive pathways have lead to a conceptual entanglement, summarized in the Cartesian view that painful sensations are the manifestation of essentially feedforward relay of sensory information along a unidirectional highway from nociceptors toward a passive brain. This incomplete and misguided view is surprising, for pain is highly context-dependent, especially with regards to cognitive and psychosocial processes (Edwards et al., 2016). Hence, cortical feedback is a key determinant factor in pain perception, and the predictive coding model, which argues that error signals must be minimized *via* dialog between feedback and feedforward signals, provides an ideal framework for disentangling the pain pathway knot.

Following the principles of predictive coding and active inference, the brain functions as a hierarchical generative model of the world, according to Bayesian probability, to explain sensory events based on past experience (Chanes and Barrett, 2016). The flow of information carrying prediction signals (feedback projections) travels from higher areas in the processing hierarchy toward lower areas. The difference between predictions and sensory input (“prediction error”) is sent back up the hierarchy (feedforward projections). In particular, the Embodied Predictive Interoception Coding (epic) model (Barrett and Simmons, 2015) is an active inference account of interoception that anticipates (rather than reacts to) external stimuli, with the goal of minimizing the difference in prediction error between internal hypotheses that are continuously being generated and external events in the environment. While epic has been argued to have significant implications for a wide range of cognitive and affective illnesses (Barrett and Simmons, 2015), it has not been incorporated into the mechanisms of normal and neuropathic pain, a powerfully salient sensory, affective, and cognitive experience. This novel concept has the potential to serve as a generative scientific framework for studying and understanding pain. Below, we present empirical evidence *vis-a-vis* the epic pain model and propose future experiments to test specific hypotheses related to a comprehensive functional connectivity map for pain at the mesoscale level.

## Thalamic bursts and pain: an EPIC coping mechanism?

Thalamic bursts during pain can be viewed as manifestation of a putative epic “error signal” generated for example from a poorly executed motor command leading to injury. In this context, thalamic bursts would be considered as an adaptive epic response to cope with pain in three ways: first, to propagate the error along cortical connections *via* somatosensory cortex and back to agranular limbic cortex; second, to change how the brain allocates attention to nociceptive input; third to generate immediate escape movements and safer behavior in the future.

Irregular burst patterns during pain have been characterized pre-clinically (Hains et al., 2005, 2006; Iwata et al., 2011) and clinically (Lenz et al., 1989, 1993). However, conflicting evidence suggests thalamic bursts may be positively (Lenz et al., 1989; Llinás et al., 1999; Hains et al., 2005, 2006; Iwata et al., 2011; Leblanc et al., 2016b) or negatively (Radhakrishnan et al., 1999; Kim et al., 2003; Cheong et al., 2008; Huh et al., 2012; Huh and Cho, 2013) correlated with pain. While tonic firing in wake monkeys follows a linear stimulus-response function, suggesting rate coding properties (Bushnell et al., 1993), thalamic bursts have been argued to signal changes in the environment to cortex more effectively than tonic firing (Swadlow and Gusev, 2001). Bursts correlate with potent activation of cortical circuits (Swadlow and Gusev, 2001) and augmentation of visual detection (Lesica et al., 2006), suggesting a dynamic role in sensory processing. Burst firing, however, is thought to be absent in thalamic neurons and of no useful function during normal waking behavior (Steriade, 2000), in contradiction to evidence supporting an important role in sensory transmission in the wake state (Sherman, 2001b; reviewed in Sherman, 2001a). Though burst probability is indeed low during waking, occasional bursts could possibly be evoked by synchronous afferent volleys (Steriade, 2001), such as during a prolonged pain episode. What generates thalamic bursts and how could bursts be related to epic?

## Thalamic bursts: EPIC pain response via thalamic reticular nucleus

Neuronal burst firing is generated by specific, intrinsic biophysical properties that have been described in detail *in vitro* (Krahe and Gabbiani, 2004), whereas the extrinsic and network mechanisms *in vivo* continue to be elucidated. Thalamic bursts are caused predominantly by strong GABAergic projections from the reticular thalamic nucleus (TRN), a thin layer overlaying sensory thalamus. Taking the epic pain model into consideration, TRN receives strong input from agranular cortical areas. Indeed, pathways for emotion and attention have been shown to converge on TRN (Zikopoulos and Barbas, 2012).

**Figure 1:**
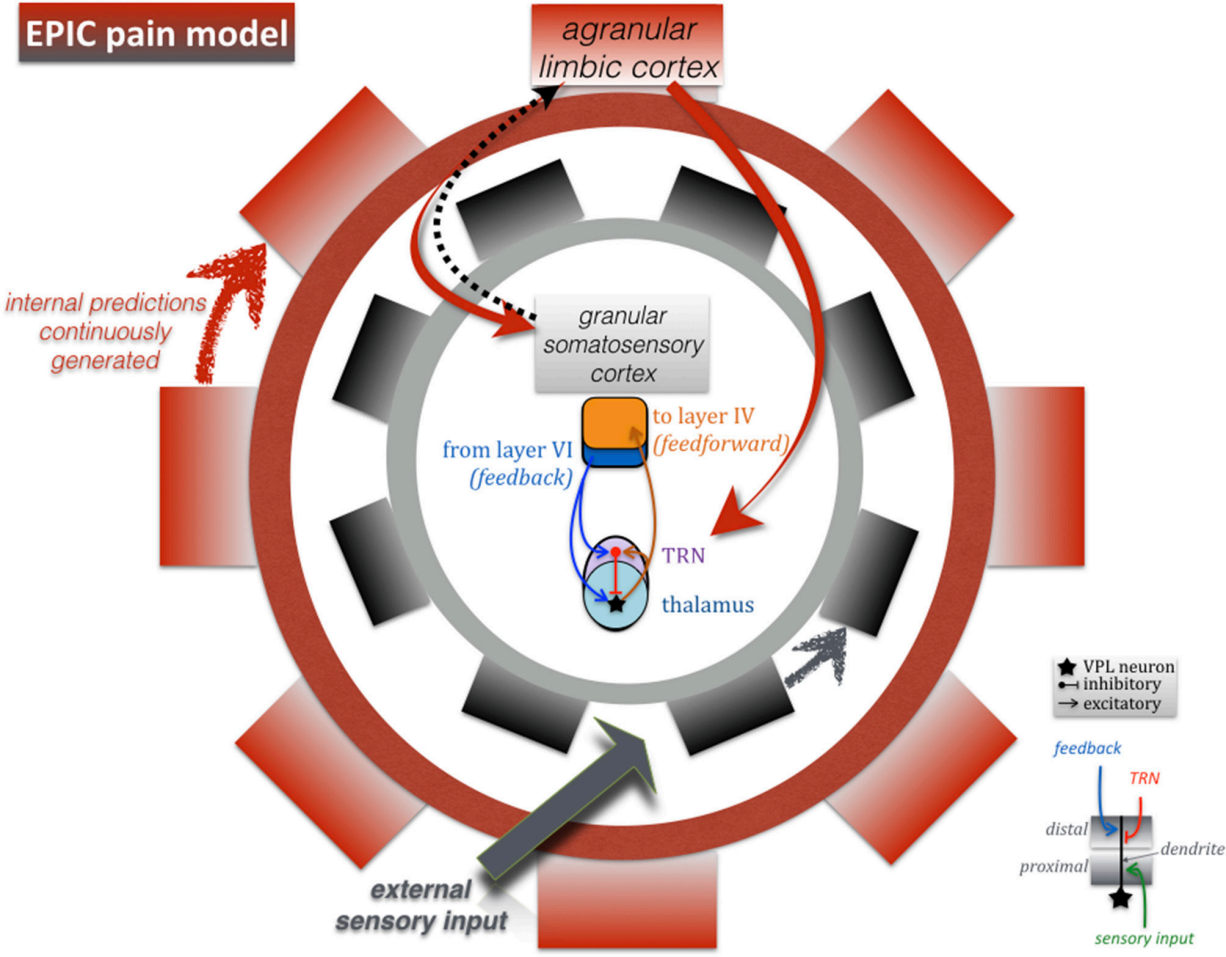
**Wheel within a wheel: the **epic** pain model**. Schematic representation of functional connectivity between the major sensory pathway (thalamus and somatosensory granular cortex), and agranular cortical areas which are continuously engaged in generating internal predictions based on interoceptive inference, even in the absence of external sensory input. The farther away an external sensory stimulus falls from the internal prediction (i.e., a powerfully salient, unexpected or “unexplained” painful event), the larger the prediction “error signal.” The objective of this feedback-feedforward dialog (which we refer to as the epic pain model) is to coordinate optimal cortical responses along two putative functional connectivity patterns: thalamic bursts via TRN, and bidirectional communication between agranular cortex (limbic/paralimbic) and granular cortex (somatosensory). According to this model, the error signal is rectified when internal predictions are updated with new information about the environment or, in the case of chronic pain, when cortical mechanisms lead to successful coping behaviors.

Thalamus and cortex form mutually interdependent structures whose coordinated actions shape the sensory experience. Thalamocortical neurons, the obligatory relay of all sensory information (except olfaction) fire in two dynamic and state-dependent modes: tonic and burst discharges of action potentials (Sherman, 2001a). The respective roles of these modes in gating sensory processing remains controversial (Sherman, 2001b; Steriade, 2001). Thalamic feedforward and cortical feedback projections both pass through, and send collaterals to, TRN neurons. In turn, TRN neurons send inhibitory projections unto thalamocortical relay neurons (Pinault, 2004), causing transient membrane hyper-polarization, de-inactivation of T-type calcium channels and bursts (Jahnsen and Llinás, 1984a,b; Figure 1). It has been suggested that TRN prevents “sensory overload” by allocating attention to relevant sensory stimuli. Thus, TRN is referred to as “guardian of the sensory gate” (Crick, 1984) and a “modality gate” (Crick, 1984; Yen and Shaw, 2003; Yen and Lu, 2013) that inhibits tactile input while allowing passage of nociceptive input to thalamus. Pharmacologic and molecular data further suggest that GABA-mediated inhibition in VPL is suppressed under pain conditions (Lee et al., 1994; Ferreira-Gomes et al., 2006) (presumably due to the inhibition of TRN neurons Peschanski et al., 1980; Yen and Shaw, 2003). This scenario is thought to lead to hyper-excitability, sensitization, and enhanced bursting of thalamic neurons. Imaging and biochemical studies in humans further support the notion that TRN neurons are inhibited during chronic pain (Henderson et al., 2013; Gustin et al., 2014; Alshelh et al., 2016; Henderson and Di Pietro, 2016). However, there is latent contradiction in the assumption that thalamic bursts are increased while TRN activity is simultaneously suppressed. A possible way to resolve this issue is to use paired thalamocortical recordings while generating thalamic bursts by selective optogenetic drive of TRN neurons (on-going experiments in the Saab lab; see future experiments).

## Cortical theta: EPIC pain response via dialog between granular-agranular cortex

The Saab lab reported in a series of studies that various pain states in rodents correlate with increased theta (4–8 Hz) oscillations in somatosensory cortex using local field potential (Leblanc et al., 2016b), electrocorticography (Leblanc et al., 2014), and electroencephalography (Leblanc et al., 2016a) recordings. Moreover, the pain-induced increase in cortical theta power is reversed upon treatment with analgesics including pregabalin and mexiletine (Leblanc et al., 2016a). Hence, our team and others have speculated that somatosensory cortical theta is a neural signature of pain in rodents (Leblanc et al., 2016a) and humans (Stern et al., 2006; Pinheiro et al., 2016). The origin of theta oscillations, however, remains elusive. We reported that functional connectivity between thalamus and somatosensory cortex is attenuated in rodent models of acute and neuropathic pain (Leblanc et al., 2014), suggesting thalamus is an *unlikely* generator of the pain-induced cortical theta (however, see Sarnthein and Jeanmonod, 2008).

Here again, applying the epic pain model, we have observed that functional connectivity between prefrontal cortex and somatosensory cortex in the rat is enhanced during neuropathic pain (Leblanc et al., 2016a). Prefrontal cortex is agranular in rat (Leonard, 2016) and thus conceivably engaged in an epic bi-directional communication with somatosensory cortex. In humans, interestingly, functional connectivity is *decreased* between Fz and Cz EEG electrodes corresponding to prefrontal and somatosensory cortex, respectively, during moderate pain (Levitt et al., 2016) noting that primate prefrontal cortex and somatosensory cortex are both granular (hence no or little communication flow is expected between these structures based on the epic pain model). Therefore, in addition to the putative epic response at a subcortical level *via* TRN, traffic patterns between agranular and granular cortices embody cortical feedback and feedforward communication from limbic/paralimbic areas to somatosensory cortex and back in a top-down and bottom-up manner, arguably generating theta oscillations.

## Future directions

Hypotheses proposed above based on the conceptual epic model of pain can be tested empirically. Deep understanding of the dynamic interactions in the thalamocortical and cortico-cortical networks, which give rise to integrated functional states including pain, is best achieved in the context of the whole organism and its behavior. Suboptimal experimental conditions imposed by pharmacological, electrical stimulation, and lesion approaches in the past have precluded a reliable inference to “causality” between thalamic bursting, cortical state, and pain. Paired, multiunit thalamocortical recordings, as well as paired, laminar cortico-cortical recordings will be necessary to directly test functional connectivity (for example coherence and phase-amplitude coupling) and directional flow of information (such as Granger causality) between brain networks at a temporal resolution high enough to resolve the neural dynamics in the TRN-thalamocortical network. Ideally, these experiments would be conducted *in vivo* during awake, freely-behaving states concomitant to intervention techniques with unprecedented selectivity (i.e., optogenetics, see Park et al., 2015; Copits et al., 2016) while longitudinally assessing the development of maladaptive pain behaviors.

Our suggested model pertains to pain in general. For acute nociceptive pain, the error signal is rectified upon elimination of the primary cause of pain. In the case of chronic pain, for example neuropathic pain secondary to peripheral nerve injury, the error signal that persists might lead an individual to engage in a multitude of behavioral modifications, some being ineffective or maladaptive. Under such neuropathic conditions, Bayesian inference systematically fails to make accurate sensory predictions due to stochastic and/or excessive nociceptive signals emanating from the injured nerve, thus contributing to symptoms of hypersensitivity such as allodynia and hyperalgesia. We acknowledge, furthermore, that chronic pain induces structural and functional reorganization of brain connectivity patterns and chronic pain *per se* has been described as evolving according to a multiphasic continuum (Baliki and Apkarian, 2015). We argue, however, that thalamic bursting and cortical theta represent hallmark neural signatures of pain irrespective of its temporal progression. A formalized computational model might further illuminate the longitudinal and quantitative relationships between the different components of the epic pain model. Noting several caveats of non-invasive electrophysiological approaches including EEG (notably poor spatial resolution and source localization, especially with respect to neural folding and volume conduction), integration of field potential recordings with resting state imaging data is key to building such biophysically-principled computational models.

## Sensation and affect are interconnected in the brain

Sensory cortical regions are anatomically connected with limbic and paralimbic cortical regions that are responsible for allostatic control, or regulation of the physiological systems of the body (Mesulam and Mufson, 1982; Mufson and Mesulam, 1982). The sensory consequences of that regulation (referred to as interoception) are experienced as low dimensional properties of affect (i.e., valence and arousal). Stimuli that evoke interoceptive changes, and therefore changes in affective experience, routinely engage sensory input regions of cortex (Barrett and Bliss-Moreau, 2009; Barbas, 2015; Barrett, 2017). A recent meta-analysis of brain imaging studies concluded that stimuli that evoke affective changes evoke sensory activations in a modality-specific manner (Satpute et al., 2015). These observations indicate that sensory areas in the brain, including somatosensory cortex, contribute to the affective experience beyond merely encoding features related to the localization and discrimination of nociceptive stimuli (Uhelski et al., 2012; Hu et al., 2014). They also dovetail with the principles of predictive coding, active inference and epic.

Current view that the sensory system is passively waiting for external sensory inputs is untenable, while pain researchers continue to rely mostly on spinal reflex behaviors to assess pain. The epic model for pain provides an alternative, more plausible explanation for a context-dependent pain experience modulated by dynamic brain states (i.e., *networks within networks* Gary Marcus, 2015) that reflect on-going cognitive and psychosocial processes. Pain does not equate to an evoked response to a noxious stimulus (see Williams and Craig, 2016 regarding the need to update the definition of pain), and the brain is a not a hollow drum struck by random sensory stimuli, rather it generates continuous predictions pertaining to the environment in order to minimize the element of surprise and maladaptive responses. To understand pain is to appreciate the state-dependent and dynamic traffic patterns in the brain.

## Summary

Thalamic neurons fire irregular bursts during neuropathic pain, a common neurologic condition characterized with sensory and affective symptoms that are poorly managed clinically. Conflicting hypotheses regarding the role of thalamic bursts in pain have been proposed. In this opinion letter, we discuss the Embodied Predictive Interoception Coding (epic) model of pain as a unifying framework for formulating testable hypotheses regarding the relation between thalamic burst and pain, and as a putative cortical feedback mechanism mediating context-dependent pain experiences and coping behaviors.

## Author contributions

CS and LB wrote the paper. CS generated the figure.

## Funding

CS was funded by investigator-initiated awards from Asahi Kasei Pharma and Boston Scientific; LB was funded by NIH, National Institute on Aging R01 AG030311, and National Cancer Institute U01 CA193632.

### Conflict of interest statement

The authors declare that the research was conducted in the absence of any commercial or financial relationships that could be construed as a potential conflict of interest.
